# Supplementary material for: A Critical View of the Application of the APEX Software (Aqueous Photochemistry of Environmentally-Occurring Xenobiotics) to Predict Photoreaction Kinetics in Surface Freshwaters
Source: Molecules. 2019 Dec 18;25(1):9. doi: 10.3390/molecules25010009 (PMC7017383; doi:10.3390/molecules25010009)
Supplement: Supplementary file 1 [file molecules-25-00009-s001.zip › 3.2.4_gcc-4.4.0.zip!/3.2.4_gcc-4.4.0/3.2.4_gcc-4.4.0/doc/octave/html/interpreter/A-Sample-Variable-Description.html]

A Sample Variable Description - Untitled


Previous: A Sample Command Description,
Up: Format of Descriptions


---

##### 1.3.5.3 A Sample Variable Description

A variable is a name that can hold a value. Although any variable
can be set by the user, built-in variables typically exist
specifically so that users can change them to alter the way Octave
behaves (built-in variables are also sometimes called user
options). Ordinary variables and built-in variables are described
using a format like that for functions except that there are no
arguments.

Here is a description of the imaginary variable
`do_what_i_mean_not_what_i_say`.

— Built-in Variable: **do\_what\_i\_mean\_not\_what\_i\_say**  
> If the value of this variable is nonzero, Octave will do what you
> actually wanted, even if you have typed a completely different and
> meaningless list of commands.

Other variable descriptions have the same format, but `Built-in
Variable' is replaced by `Variable', for ordinary variables, or
`Constant' for symbolic constants whose values cannot be changed.
